# Gigantol inhibits Wnt/β-catenin signaling and exhibits anticancer activity in breast cancer cells

**DOI:** 10.1186/s12906-018-2108-x

**Published:** 2018-02-14

**Authors:** Shubin Yu, Zhongyuan Wang, Zijie Su, Jiaxing Song, Liang Zhou, Qi Sun, Shanshan Liu, Shiyue Li, Ying Li, Meina Wang, Guo-Qiang Zhang, Xue Zhang, Zhong-Jian Liu, Desheng Lu

**Affiliations:** 1 Guangdong Key Laboratory for Genome Stability & Disease Prevention, Cancer Research Center, Department of Pharmacology, Shenzhen University Health Science Center, Shenzhen, 518060 China; 2 Shenzhen Key Laboratory for Orchid Conservation and Utilization, The National Orchid Conservation Center of China and The Orchid Conservation and Research Center of Shenzhen, Shenzhen, 518114 China; 3 School of Traditional Chinese Materia Medica, Shenyang Pharmaceutical University, Shenyang, 110016 China

**Keywords:** Gigantol, LRP6, Wnt/β-catenin signaling, Breast cancer, Anticancer activity

## Abstract

**Background:**

Gigantol is a bibenzyl compound derived from several medicinal orchids. This biologically active compound has been shown to have promising therapeutic potential against cancer cells, but its mechanism of action remains unclear.

**Methods:**

The inhibitory effect of gigantol on Wnt/β-catenin signaling was evaluated with the SuperTOPFlash reporter system. The levels of phosphorylated low-density lipoprotein receptor related protein 6 (LRP6), total LRP6 and cytosolic β-catenin were determined by Western blot analysis. The expression of Wnt target genes was analyzed using real-time PCR. Cell viability was measured with a MTT assay. The effect of gigantol on cell migration was examined using scratch wound-healing and transwell migration assays.

**Results:**

Gigantol decreased the level of phosphorylated LRP6 and cytosolic β-catenin in HEK293 cells. In breast cancer MDA-MB-231 and MDA-MB-468 cells, treatment with gigantol reduced the level of phosphorylated LRP6, total LRP6 and cytosolic β-catenin in a dose-dependent manner, resulting in a decrease in the expression of Wnt target genes Axin2 and Survivin. We further demonstrated that gigantol suppressed the viability and migratory capacity of breast cancer cells.

**Conclusion:**

Gigantol is a novel inhibitor of the Wnt/β-catenin pathway. It inhibits Wnt/β-catenin signaling through downregulation of phosphorylated LRP6 and cytosolic β-catenin in breast cancer cells.

## Background

The Wnt/β-catenin signaling pathway plays a crucial role in embryonic development and tumorigenesis [1, 2]. In the absence of Wnt proteins, β-catenin is constantly degraded by a destruction complex containing the scaffold protein Axin, the adenomatosis polyposis coli (APC) protein, and the enzyme glycogen synthase kinase-3β (GSK3β). The Wnt/β-catenin signaling cascade is initiated when the secreted Wnt proteins bind to the Wnt coreceptor LRP5/6 and a member of the Frizzled (Fzd) family. Subsequently, the adaptor protein Dishevelled (DVL) is phosphorylated, which triggers the disruption of the destruction complex and prevents degradation of β-catenin in the proteasome. Stabilized β-catenin accumulates in the cytoplasm and translocates into the nucleus to form a complex with transcription factors of the T-cell factor/lymphoid enhancing factor (TCF/LEF) family to activate transcription of Wnt target genes [2, 3]. Compelling evidence has indicated that aberrant activation of Wnt/β-catenin signaling contributes to the initiation, progression and metastasis in various human cancers, including breast cancer [1, 4–10]. There is an urgent need to develop drugs directed at mediating various components of the Wnt/β-catenin signaling pathway.

Gigantol is a bibenzyl-type phenolic compound isolated from several medicinal orchids. This biologically active compound has been shown to have significant antioxidative [11–13], antispasmodic [3, 14], antinociceptive [15], anti-inflammatory [15, 16], anti-platelet aggregative [17], and anticancer properties. Gigantol has been shown to exhibit significant anticancer activity against several lines of cancer cells [18–22]. In lung cancer cells, gigantol suppresses cell proliferation, migration, epithelial to mesenchymal transition (EMT) and cancer stem cell (CSC) features [18–21]. Moreover, gigantol has been shown to inhibit and induce apoptosis of HepG2 cells via the PI3K/Akt/NF-κB signaling pathway [22]. In the present study, our results demonstrated that gigantol is a novel inhibitor of Wnt/β-catenin signaling. Gigantol inhibited Wnt/β-catenin signaling through downregulation of phosphorylated LRP6 and cytosolic β-catenin in breast cancer cells.

## Methods

### Materials

**Fig. 1:**
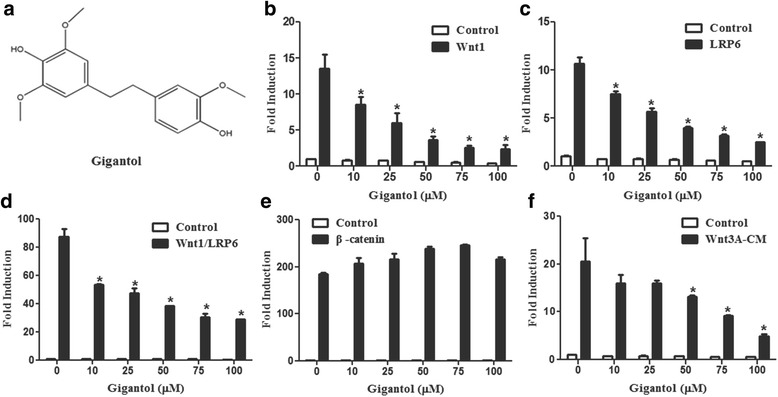
Gigantol suppresses the activity of the Wnt signaling pathway. **a** Structure of gigantol. **b**-**e** HEK293T cells were transfected with SuperTopFlash reporter gene together with control vector or expression plasmids encoding Wnt1 (**b**), LRP6 (**c**), Wnt1 and LRP6 (**d**), and β-catenin (**e**). After transfection for 24 h, the transfected cells were treated with DMSO or the indicated concentrations of gigantol for 24 h (**f**). The SuperTopFlash reporter gene was transfected into HEK293T cells. At 24 h after transfection, cells were incubated with the control or Wnt3A-CM containing DMSO or the specified concentrations of gigantol for 24 h. The luciferase values were normalized using β-gal activities. Statistical significance between groups is calculated by two-way ANOVA, *P < 0.05 compared to cells transfected with Wnt1, LRP6, or Wnt1 and LRP6, or incubated with Wnt3A-CM containing DMSO

Gigantol (Fig. 1a) was purchased from Shanghai Yuanye Biotechnology Company. The reporter plasmids SuperTopFlash, NFAT-Luc and AP1-Luc and the expression plasmids encoding Wnt1, LRP6, β-catenin, NFATc, H-ras^v12^ and β-gal have been described previously [23].

### Cell culture

The human embryonic kidney HEK293T cells, mouse fibroblast L-cells, and L-cells stably expressing Wnt3A (L-Wnt3A) were grown in Dulbecco’s modified Eagle’s media (DMEM) containing 10% fetal bovine serum (FBS), 100 U/mL penicillin, and 100 μg/mL streptomycin in 5% CO_2_ at 37 °C. Human breast cancer MDA-MB-231 and MDA-MB-468 cells were cultured in Leibovitz’s L-15 medium (Gibico) supplemented with 10% FBS, 100 U/mL penicillin, and 100 μg/mL streptomycin at 37 °C without CO_2_. Non-tumorigenic human mammary epithelial MCF-10A cells were maintained in DMEM/F12 medium containing 5% horse serum, 10 mg/mL insulin, 20 ng/mL epidermal growth factor (EGF), 100 mg/mL choleratoxin, 0.5 mg/mL hydrocortisone, 100 U/mL penicillin, and 100 μg/mL streptomycin and incubated in 5% CO_2_ at 37 °C.

### Transfection and luciferase analyses

HEK293T cells were grown in 24-well plates for 16–20 h and then transfected with 250 ng of reporter plasmid, 50 ng of pCMXβgal plasmid, and 50–200 ng of the indicated expression plasmids using TransIn EL Transfection Reagent (Transgene) according to the manufacturer’s instructions. At 24 h after transfection, cells were incubated with either DMSO or various indicated concentrations of gigantol. The control conditioned medium and Wnt3A conditioned medium (Wnt3A-CM) were collected as previously described [24]. Luciferase activity was detected using a luciferase assay kit (Promega) according to the manufacturer’s instructions and the resulting luciferase values were normalized to β-gal activities to control for transfection efficiency.

### Preparation of cytosolic extracts for β-catenin detection

Cells were first seeded onto a 6-well plate. After overnight incubation, cells were treated with various concentrations of gigantol for 24 h. Cells were then lysed with 0.015% digitonin in PBS supplemented with 2.5 mM sodium pyrophosphate, 1 mM β-glycerol phosphate, 1 mM sodium orthovanadate, 2 μg/mL leupeptin, and 1 mM PMSF. After being centrifuged at 3000 rpm for 3 min, the supernatant was collected as the cytosolic extracts.

### Western blot analyses

Cell lysates were prepared with buffer containing 20 mM Tris·HCl (pH 7.4), 150 mM NaCl, 1 mM EDTA, 1 mM EGTA, 1% Triton X-100, 2.5 mM sodium pyrophosphate, 1 mM β-glycerol phosphate, 1 mM sodium orthovanadate, 2 μg/mL leupeptin, and 1 mM PMSF. Equal amounts of protein from each sample were separated by SDS-PAGE followed by immunobloting with the following antibodies: anti-LRP6 (Cell Signaling Technology), anti-phosphorylated LRP6 (Ser1490) (Cell Signaling Technology), anti-β-catenin (Santa Cruz Biotechnology), and anti-GAPDH (Transgene).

### Quantitative real-time PCR analyses

Total RNA was extracted using RNAiso Plus (TaKaRa) and subsequently reverse-transcribed into cDNA using the Primescript RT Reagent Kit (TaKaRa) according to the manufacturer’s instructions. Quantitative PCR analysis was performed with FastStart Universal SYBR Green Master (Roche). The primers were as follows: Axin2: sense, 5’-TACACTCCTTATT-GGGCGATCA-3′; antisense, 5’-TTGGCTACTCGTAAAGTTTTGGT-3′; Survivin: sense, 5′- AGGACCACCGCATCTCTACAT-3′; antisense, 5’-AAGTCTGG-CTCGTTCTCAGTG-3′; GAPDH: sense, 5’-CCAGAACATCATCCCTGCCTCTACT-3′; antisense, 5′-GGTTT-TTCTAGACGGCAGGTCAGGT-3′.

### Cell viability assays

Cells (1 × 10^4^ cells per well) were seeded onto 96-well plates and incubated overnight. Cells were then treated with DMSO or various concentrations of gigantol for 48 h before being cultured with fresh medium containing MTT (5 mg/mL) for another 4 h. The formazan crystals were dissolved in DMSO and the absorbance of the formazan solution measured at 570 nm using a microplate reader.

### Wound-healing assays

The MDA-MB-231 cells were seeded onto a 12-well plate and a pipette tip was used to scratch the center of monolayer. Cells were then incubated with fresh medium containing DMSO or 25, 50, 100 μM of gigantol for 24 h and then photomicrographed.

### In vitro migration analyses

As previously described [25], cells (2 × 10^5^) were trypsinised and resuspended in serum-free medium and then seeded onto 24-well transwell chambers with 8-μm pore membrane in 100 μL serum-free medium contained DMSO or various concentrations of gigantol. The lower chamber contained medium supplemented with 20% FBS. After incubation for 6 h, the unmigrated cells on the upper side of membrane were removed with a cotton swab and the migrated cells stained with crystal violet and photomicrographed.

### Statistical analyses

Statistical analyses were performed using two-way ANOVA GraphPad Prism 5.0. Results are presented as mean ± SD. Differences at *P* < 0.05 were considered statistically significant.

## Results

### Gigantol inhibits Wnt/β-catenin signaling in HEK293 cells

We explored the effects of some natural products on Wnt/β-catenin signaling using the SuperTOPFlash cell reporter system. Gigantol exhibited an inhibitory effect on Wnt reporter gene expression. To confirm its inhibitory effect on Wnt/β-catenin signaling, the SuperTopFlash reporter was transfected into HEK293 cells along with Wnt1, LRP6, Wnt1/LRP6, or β-catenin expression plasmids. Gigantol treatment inhibited Wnt signaling activated by Wnt1 (Fig. 1b), LRP6 (Fig. 1c) and Wnt1/LRP6 (Fig. 1d) in a dose-dependent manner. Moreover, the increased SuperTopFlash activity induced by the Wnt3A-conditioned medium (Wnt3A-CM) was dose-dependently blocked by gigantol (Fig. 1f). However, gigantol could not suppress Wnt signaling activated by β-catenin (Fig. 1e), suggesting that gigantol may act on the upstream elements of the pathway.

### Gigantol has no inhibitory effect on the NFAT and AP-1 signaling pathways

**Fig. 2:**
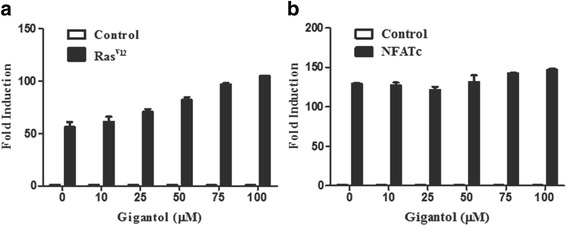
Gigantol does not inhibit the luciferase activity of NFAT or the AP1 reporter gene. **a** A NFAT-Luc reporter along with a control vector or expression plasmid encoded NFATc were transfected into HEK293T cells. **b** An AP1-Luc reporter together with control vector or a constitutively active Ras^v12^ expression plasmid were transfected into HEK293T cells. Gigantol treatment and luciferase activity are presented in Fig. 1

To test the effect of gigantol on other signaling pathways, transfection assays were also performed using NFAT-Luc and AP1-Luc reporters. The expression plasmids encoding NFATc and Ras^v12^ were used to activate the NFAT and AP1 signaling pathways in HEK293 cells, respectively. As shown in Fig. 2, gigantol at Wnt inhibitory concentrations could not suppress the activity of the NFAT-Luc and AP1-Luc reporters (Fig. 2a-b).

### Gigantol suppresses LRP6 phosphorylation induced by Wnt1 or Wnt3A-CM

**Fig. 3:**
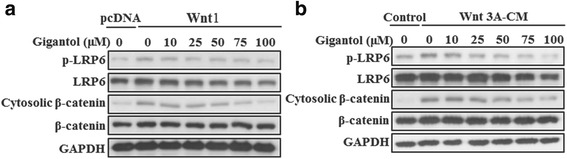
Gigantol inhibits the Wnt/β-catenin signaling cascade in HEK293T cells. **a** The expression plasmid encoding Wnt1 was transfected into HEK293T cells. The cells were then incubated with the indicated concentrations of gigantol for 24 h. **b** HEK293T cells were treated with the control medium, Wnt3A-CM containing DMSO or the indicated concentrations of gigantol for 24 h. Phosphorylated LRP6, total LRP6, cytosolic β-catenin and total β-catenin were detected by immunoblotting

To explore the molecular mechanism underlying Wnt signaling inhibition by gigantol, we examined the effect of gigantol on LRP6 phosphorylation. As expected, Wnt1 expression or specifically Wnt3A-CM induced endogenous LRP6 phosphorylation at serine 1490 and increased cytosolic β-catenin in HEK293 cells (Fig. 3a-b). Treatment with gigantol noticeably blocked Wnt1- or Wnt3A-induced LRP6 phosphorylation, leading to a decrease in cytosolic β-catenin levels (Fig. 3a-b). These results indicate that gigantol may inhibit Wnt/β-catenin signaling by targeting LRP6.

### Gigantol suppresses Wnt/β-catenin signaling in breast cancer cells

**Fig. 4:**
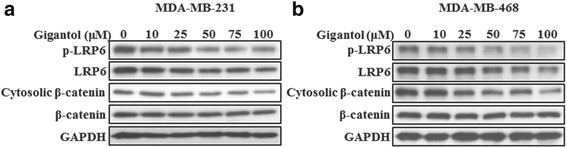
Gigantol suppresses Wnt/β-catenin signaling pathway in breast cancer cells. **a-b** MDA-MB-231 (**a**) and MDA-MB-468 (**b**) cells were treated with the indicated concentrations of gigantol for 24 h. Phosphorylated LRP6, total LRP6, cytosolic β-catenin, and total β-catenin were detected by immunoblotting

The MDA-MB-231 and MDA-MB-468 cell lines were used to assess the effect of gigantol on Wnt/β-catenin signaling in breast cancer cells. In both cell lines, gigantol markedly decreased the levels of phosphorylated LRP6 and total LRP6 dose-dependently (Fig. 4a-b). Furthermore, we observed a significant decrease in cytosolic β-catenin after gigantol treatment (Fig. 4a-b), indicating that gigantol inhibits Wnt/β-catenin signaling in breast cancer cells by suppressing LRP6 expression.

### Gigantol down-regulates expression of Wnt target genes in breast cancer cells

**Fig. 5:**
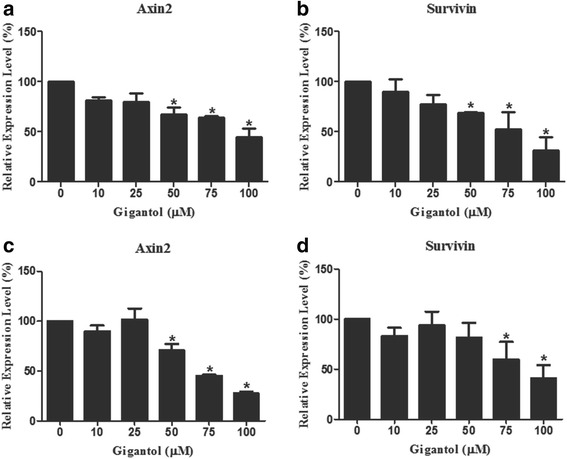
Gigantol reduces the mRNA levels of Wnt target genes in breast cancer cells. **a-d** MDA-MB-231 (**a** and **b**) and MDA-MB-468 (**c** and **d**) cells were treated with the specified amounts of gigantol for 24 h. RNA was extracted and then reverse-transcribed into cDNA. Prepared cDNA was then subjected to real-time PCR analysis to detect the mRNA expression of Axin2 (**a** and **c**) and Survivin (**b** and **d**). Gigantol treatment significantly reduced the mRNA level of Axin2 and Survivin compared with DMSO treated control cells, * *p* < 0.05

To further confirm the inhibitory effects of gigantol on Wnt/β-catenin signaling in breast cancer cells, we investigated the effect of gigantol on the expression of Axin2 and Survivin, both of which are well-known target genes in the Wnt/β-catenin pathway. The results of real-time PCR showed that mRNA expression of Axin2 and Survivin was significantly decreased after gigantol treatment in breast cancer MDA-MB-231 and MDA-MB-468 cells (Fig. 5a-b).

### Gigantol inhibits viability and migration of breast cancer cells

**Fig. 6:**
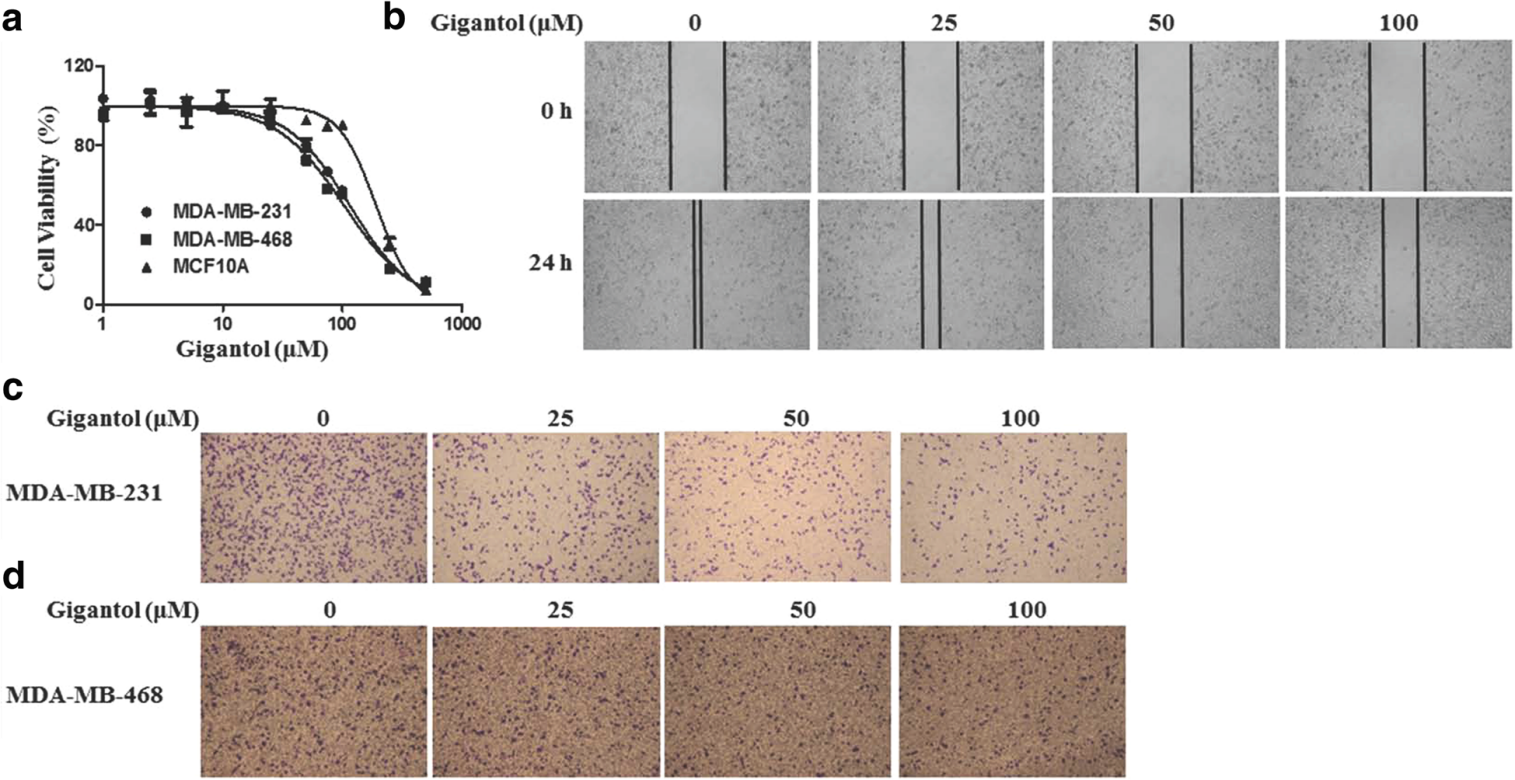
Effect of gigantol on viability and migration of breast cancer cells. **a** MDA-MB-231, MDA-MB-468 and MCF10A cells were treated with the indicated concentrations of gigantol for 48 h, respectively. Cell viability was assessed with the MTT assay. **b** MDA-MB-231 cells were grown in medium contained 10% FBS. The cell monolayers were scratched and treated with varying concentrations of gigantol for 24 h and then photomicrographed. **c-d** MDA-MB-231 (**c**) or MDA-MB-468 (**d**) cells were treated with the indicated concentrations of gigantol for 6 h in a transwell assay. Cells that migrated through transwells were stained with crystal violet and photomicrographed

To assess the potential cytotoxic effects of gigantol on breast cancer cells, MDA-MB-231 and MDA-MB-468 cells were treated with varying concentrations of gigantol ranging from 1.25 to 500 μM for 48 h. Gigantol reduced the viability of breast cancer cells, with IC_50_ values of 115.2 ± 6.7 μM in MDA-MB-231 cells and 103.6 ± 10.9 μM in MDA-MB-468 cells (Fig. 6a). In contrast, the IC_50_ value for the non-tumorigenic human mammary epithelial MCF10A cells was 192.1 ± 17.0 μM, indicating that gigantol has selective cytotoxicity in cancer cells.

We further tested the effect of gigantol on cell migration with wound-healing and transwell assays. As shown in Fig. 6b, gigantol dose-dependently decreased the migration of MDA-MB-231 cells to the scratched area compared to untreated control cells (Fig. 6b). Consistently, a similar trend was observed in transwell assays. Treatment with gigantol suppressed the migration of breast cancer MDA-MB-231 (Fig. 6c) and MDA-MB-468 cells (Fig. 6d).

## Discussion

Gigantol has been shown to have promising therapeutic potential in cancer cells [18–22], but its mechanism of action remains unclear. In the present study, we identified gigantol as a novel small-molecule Wnt/β-catenin inhibitor. Gigantol inhibited LRP6 phosphorylation and Wnt/β-catenin signaling activation induced by Wnt1 or Wnt3A-CM in HEK293 cells. In breast cancer cells, gigantol treatment significantly decreased the levels of phosphorylated LRP6, total LRP6 and cytosolic β-catenin, resulting in a decrease in the expression of Wnt target genes Axin2 and Survivin. Moreover, gigantol-induced antagonism of Wnt/β-catenin signaling occurs at comparable concentrations with those required to suppress viability and migration of breast cancer cells. These results suggest that the anticancer activity of gigantol is associated with its inhibitory effects on Wnt/β-catenin signaling.

LRP6 is an essential coreceptor in the Wnt/β-catenin signaling pathway and thus a potential therapeutic target in breast cancer treatments. Several studies have shown that LRP6 is up-regulated in human breast cancer cells [6]. In MMTV-LRP6 transgenic mice, overexpression of LRP6 in the mouse mammary gland is sufficient to activate Wnt/β-catenin signaling and induce mammary hyperplasia [26]. LRP6 silencing in breast cancer cells reduced Wnt signaling, cell proliferation, and in vivo tumor growth [27]. The LRP6 antagonist Mesd markedly suppressed tumor growth in MMTV-Wnt1 xenograft models. Moreover, specific LRP6 antibodies were able to block growth of MMTV-Wnt1 or MMTV-Wnt3 xenografts in vivo [28–30]. Multiple small molecule LRP6 inhibitors, such as salinomycin [31], prodigiosin [23], niclosamide [32], silibinin [33] and rottlerin [34], have been identified to exert anticancer activity in breast cancer cells. Our results indicate that the anti-breast cancer activity of gigantol is associated with its inhibition of LRP6 activity.

A recent study showed that gigantol inhibited proliferation and induced apoptosis of liver cancer HepG2 cells [22]. In non-small cell lung cancer H460 cells, gigantol reduced the viability of cancer cells with IC_50_ values of 247.55 ± 4.94 μM [18]. Gigantol also inhibited the migration of lung cancer cells and induced lung cancer cell apoptosis through a mitochondria-dependent pathway [19]. Unahabhokha et al. reported that gigantol attenuated EMT via AKT downregulation [35, 36]. Interestingly, it was noted that gigantol could decrease β-catenin expression in lung cancer cells [36], which is consistent with its inhibitory role in the Wnt/β-catenin signaling pathway. Furthermore, gigantol decreased stemness in the lung cancer cells and reduced well-known lung CSC markers, including CD133 and ALDH1A1 [20]. Considering the importance of Wnt/β-catenin signaling in stem cell self-renewal and various malignancies, the Wnt antagonistic action of gigantol may contribute to its CSC suppressing activity. Our results demonstrated that gigantol has the ability to suppress the viability and migration of breast cancer cells. Since CSCs play such a crucial role in the recurrence, metastasis, and drug resistance of breast cancer, it will be interesting to investigate the inhibitory action of gigantol on breast CSCs.

## Abbreviations

APC: Adenomatosis polyposis coli
CSCs: Cancer stem cells
DMEM: Dulbecco’s modified Eagle’s media
DVL: Dishevelled
EGF: Epidermal growth factor
EMT: Epithelial to mesenchymal transition
FBS: Fetal bovine serum
Fzd: Frizzled
GSK3β: Glycogen synthase kinase-3β
LRP5/6: Low-density lipoprotein receptor-related protein5/6
TCF/LEF: T-cell factor/lymphoid enhancing factor
Wnt3A-CM: Wnt3A conditioned medium
